# Methicillin-resistant *Staphylococcus aureus*, Western Australia

**DOI:** 10.3201/eid1110.050125

**Published:** 2005-10

**Authors:** Lynne Dailey, Geoffrey W. Coombs, Frances G. O'Brien, John W. Pearman, Keryn Christiansen, Warren B. Grubb, Thomas V. Riley

**Affiliations:** * Curtin University of Technology, Bentley, Western Australia, Australia; † Royal Perth Hospital, Perth, Western Australia, Australia; ‡ The University of Western Australia and Western Australian Centre for Pathology & Medical Research, Nedlands, Western Australia, Australia

**Keywords:** Staphylococcus aureus, community-associated MRSA, epidemiology, surveillance, multi-drug resistance, research

## Abstract

Methicillin-resistant *Staphylococcus aureus* (MRSA) continues to be a notable cause of hospital-acquired infections. A statewide screening and control policy was implemented in Western Australia (WA) after an outbreak of epidemic MRSA in a Perth hospital in 1982. We report on statutory notifications from1998 to 2002 and review the 20-year period from 1983 to 2002. The rate of reporting of community-associated Western Australia MRSA (WAMRSA) escalated from 1998 to 2002 but may have peaked in 2001. Several outbreaks were halted, but they resulted in an increase in reports as a result of screening. A notable increase in ciprofloxacin resistance during the study period was observed as a result of more United Kingdom epidemic MRSA (EMRSA) -15 and -16. WA has seen a persistently low incidence of multidrug-resistant MRSA because of the screening and decolonization program. Non–multidrug-resistant, community-associated WAMRSA strains have not established in WA hospitals.

## Abstract 2

Endemic MRSA persists in Western Australia despite control measures.

Recent publications suggest that the epidemiology of methicillin-resistant *Staphylococcus aureus* (MRSA) infections is changing, and hospitalization is no longer necessarily a risk factor (*1**–**3*). Reports indicate that community-associated MRSA infection is now a worldwide phenomenon. A common theme in these publications is that the affected populations are usually marginalized, indigenous peoples, such as American Indians in the midwestern United States (*4**,**5*), Canadian aboriginals (*6**,**7*), and Pacific Islanders in the southwestern Pacific region (*8**,**9*). Aboriginal Australians also appear to be at higher risk for community-associated MRSA (*10*). However, overcrowding or situations where persons are in close proximity to others, such as in prisons (*11*) and on sporting teams (*12*), may represent the true risk. The increased prevalence in children (*13*) may be due to the enhanced opportunity for exposure at schools and daycare centers.

In the early 1980s, epidemic MRSA (EMRSA) first appeared on the east coast of Australia; these strains were often referred to as eastern Australian MRSA (*14*). EMRSA were multidrug-resistant, and they became endemic in many large hospitals throughout Australia, with the exception of Western Australia (WA) (*15*). The establishment of EMRSA in WA hospitals has been prevented because of a screening and control program (see Methods) and the isolation of the state (*14**,**16**,**17*). However, late in the 1980s, non–multidrug-resistant community-associated MRSA emerged in WA (*14**,**18**,**19*). MRSA isolated from patients living in the remote Kimberley region in the northern part of the state (Figure 1) were phenotypically and genotypically different from EMRSA and became known as WAMRSA (*18**,**19*). Some WAMRSA have since acquired a multidrug-resistance plasmid that encodes resistance determinants, including trimethoprim, tetracycline, and high-level mupirocin resistance (*14**,**18**,**19*). During the 1990s, WAMRSA spread to most regions of WA (*14**,**18*), and a substantial number of cases of infection and colonization occurred in metropolitan Perth by 1997 (*20*).

**Figure 1:**
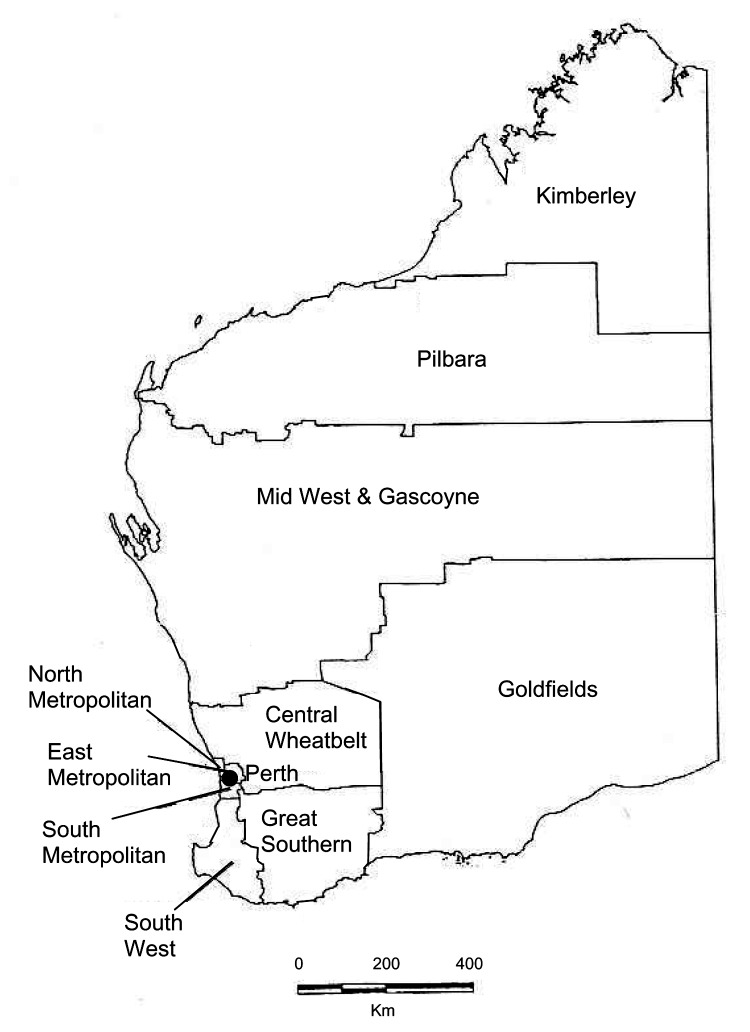
Health regions of Western Australia.

This retrospective review of statutory MRSA notification data was conducted for the period 1998 to 2002. The aim of the study was to report changes in reporting rates over time and by location, to describe the distribution by age and sex of patients, and to document temporal changes in antimicrobial resistance patterns. The findings were compared to those in previous publications that covered MRSA notification data in WA for the period 1983 to 1996 (*14**,**18**,**20*).

## Methods

### Background

A statewide screening and control policy was implemented in WA after an outbreak of EMRSA in a Perth hospital in 1982 (*16*). The policy involved screening all patients who were admitted to hospitals from different states or overseas and all new staff members who had worked outside WA in the previous 12 months (*17*). After screening, patients infected or colonized with MRSA were isolated and treated; infected or colonized staff members were prohibited from contact with patients until the organism was eradicated. In WA, MRSA infection or colonization has been a reportable condition since 1985. The WA Department of Health electronically flags cases of MRSA, which allows infected persons to be identified and isolated upon admission to any WA public hospital (*17*).

Since the screening and control policy was introduced, the identity of MRSA clinical isolates and those isolated through screening has been confirmed by a reference laboratory by using standard procedures; antimicrobial drug susceptibility was determined by Clinical and Laboratory Standards Institute (formerly NCCLS) methods (*21*). Until 1997, the reference laboratory was in the Division of Microbiology and Infectious Diseases at the WA Centre for Pathology and Medical Research; thereafter it was in the Royal Perth Hospital/Curtin University Gram Positive Bacteria Typing and Research Unit.

From 1983 to 1997, MRSA was categorized as EMRSA or WAMRSA according to antimicrobial drug resistance patterns based on previous genetic analysis (*14*). EMRSA strains were resistant to β-lactam antimicrobial drugs, gentamicin, or both erythromycin and tetracycline. Strains resistant to β-lactams only, β-lactams and erythromycin, or tetracycline but not gentamicin were classified as WAMRSA. This approach has several limitations that have been alluded to in previous publications (*14**,**18**,**20*), such as changes in susceptibility pattern as a result of plasmid acquisition. Consequently, a more sophisticated system for differentiating isolates was developed at the Royal Perth Hospital laboratory that detected the introduction into WA of UK EMRSA-15 and Irish-2 strains of MRSA (*22*).

### Data

Information on infection and colonization with MRSA was obtained from the database held by the Communicable Diseases Control Branch of the WA Department of Health. MRSA colonization was determined by screening patients, staff, and contacts by methods as described (*17*). Isolates recovered on screening and clinical isolates were sent to the Royal Perth Hospital laboratory for characterization by several procedures, including bacteriophage typing, routine antibiogram, urease production, extended antibiogram/resistogram, coagulase gene typing, and pulsed-field gel electrophoresis (*23**,**24*). Information collected with isolates included basic case demographics and details pertaining to the organism, including isolation site. In addition, whether the notification was the result of MRSA isolates found in a clinical specimen or from a screening specimen was recorded. Multiple cultures on the same patient were not included unless it had been determined that the patient was clear of MRSA colonization or infection after the process outlined (*17*).

Crude notification rates for health regions (Figure 1) were calculated with population estimates based on the 2001 census (*25*). Differences in proportions were compared by using the chi-square test, while changes over time were assessed by using chi square for trend.

## Results

**Figure 2:**
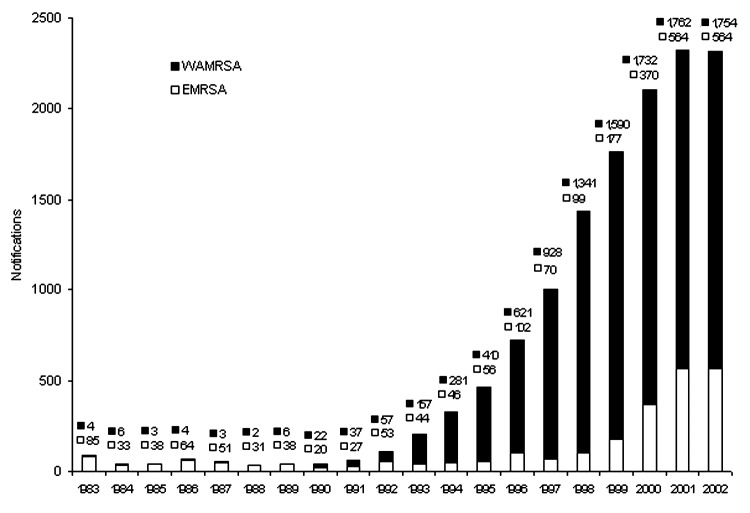
Notifications of methicillin-resistant Staphylococcus aureus (MRSA) in Western Australia (WA), 1983–2002, WAMRSA versus epidemic MRSA. Note: Not included are 4 in 2001 and 12 in 2002 of Western Samoan Phage Pattern.

From 1998 to 2002, a total of 9,955 notifications of MRSA were made in WA; 1,441 notifications were made in 1998, 1,767 in 1999, 2,102 in 2000, 2,326 in 2001, and 2,319 in 2002. Table 1 shows the numbers of notifications and crude notification rates per 100,000 population of the various health regions of WA. Of the 9,955 notifications, 9,728 gave permanent addresses within WA. The highest notification rates were recorded in the Kimberley region, followed by the East Metropolitan and Goldfields regions. The average yearly notification rate for the whole state during this period was 107.7/100,000 population. Figure 2 shows notifications of WAMRSA and EMRSA in WA from 1983 to 2002. This figure shows a marked increase in WAMRSA from 1991 to 2002 (peak), with a slowing in the notification rate after 2000. EMRSA peaked in 2001 and declined in 2002.

**Table 1:** Notifications of MRSA and rates per 100,000 population*

Location	No. notifications (rate)					Total no. MRSA
	1998	1999	2000	2001	2002	
Central Wheatbelt	13 (25.5)	28 (54.9)	34 (66.7)	37 (72.6)	41 (80.4)	153
Goldfields	112 (199.9)	110 (196.3)	83 (148.1)	91 (162.4)	56 (99.9)	452
Great Southern	60 (87.8)	55 (80.5)	43 (62.9)	87 (127.3)	91 (133.2)	336
Kimberley	107 (255.0)	97 (231.1)	140 (333.6)	112 (266.9)	115 (274.0)	571
East Metropolitan	251 (109.2)	348 (151.4)	415 (180.5)	529 (230.1)	446 (194.0)	1,989
North Metropolitan	175 (33.5)	248 (47.5)	324 (62.1)	384 (73.5)	384 (73.5)	1,515
South Metropolitan	508 (86.4)	610 (103.8)	769 (130.8)	745 (126.7)	830 (141.2)	3,462
Mid West & Gascoyne	79 (117.3)	98 (145.5)	116 (172.2)	126 (187.0)	120 (178.1)	539
Pilbara	47 (109.9)	44 (102.9)	47 (109.9)	38 (88.9)	52 (121.6)	228
South West	68 (37.4)	89 (48.9)	78 (42.9)	111 (61.0)	137 (75.3)	483
Other/Unknown	21	40	53	66	47	227
Total	1,441 (77.9)	1,767 (95.5)	2,102 (113.7)	2,326 (125.8)	2,319 (125.4)	9,995

*MRSA, methicillin-resistant *Staphylococcus aureus*.

**Figure 3:**
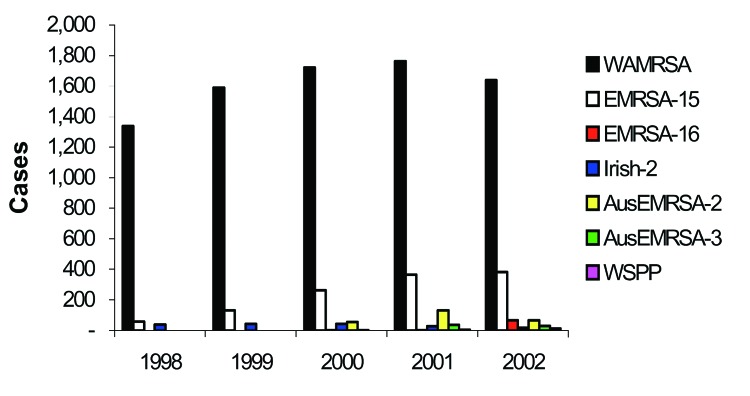
Methicillin-resistant Staphylococcus aureus (MRSA) in Western Australia, 1998–2002, by type.

The distribution of MRSA by type is shown in Figure 3. In 1998, 6.4% of MRSA notifications were classified as EMRSA, increasing to 24.4% in 2002. The greatest contributor to EMRSA was UK EMRSA-15, which rose from 55 reports in 1998 to 383 in 2002. UK EMRSA-16 increased substantially from a few notifications in 2000–2001 to 66 notifications in 2002. Irish-2 notifications remained constant early in the 5-year period at ≈40 per year but fell to 29 in 2001 and 18 in 2002. Australian EMRSA was maintained at a variable but relatively low level, except in 2001 when 131 notifications occurred. Overall, 94% of community-associated WAMRSA were classified into 3 clones: ST1-MRSA-IV (55%), ST129-MRSA-IV (30%), and ST5-MRSA-IV (9%). Of the community-associated WAMRSA, 97% were staphylococcal chromosome cassette (SCC) *mec* type IV and 3% were SCC*mec* type V (unpub. data). During this period, Western Samoan Phage Pattern strains were isolated occasionally.

Some of the variability in notifications was related to outbreaks and subsequent contact screenings. Overall, 75% of notifications (7,913) were due to MRSA isolates found in routine clinical specimens; 25% (1,980) were due to a survey (Table 2). The annual proportion of isolates due to screening increased significantly during the 5-year period (from 10% in 1998 to 39% in 2002) (chi square for trend 35.696, p<0.0001). Of the 1,980 MRSA detected in screening specimens, 86.7% were WAMRSA. Of the 9,889 notifications over the 5-year period where the appropriate information was provided, 93% were due to MRSA isolates found in patient specimens, however, 5% of notifications involved staff, and 2% were from other contacts (Table 3). The number of notifications due to a staff member who had MRSA increased from 73 in 2000 to 173 in 2001.

**Table 2:** Notifications of MRSA from routine and survey specimens*

Year	Routine no. (%)	Survey no. (%)	Total
1998	1,308 (90)	131 (10)	1,439
1999	1,535 (85)	228 (15)	1,763
2000	1,756 (81)	338 (19)	2,094
2001	1,661 (62)	631 (38)	2,292
2002	1,653 (61)	652 (39)	2,305
Total	7,913 (75)	1,980 (25)	9,893

*Data were not recorded for 62 specimens; *MRSA, methicillin-resistant *Staphylococcus aureus*.

**Table 3:** Notifications of MRSA in patients, staff members, and other contacts*

Type	1998	1999	2000	2001	2002	Total (%)
Patients	1,357	1,644	1,986	2,081	2,171	9,239 (93)
Staff	22	71	73	173	110	449 (5)
Other	46	43	36	52	24	201 (2)
Total	1,425	1,758	2,095	2,306	2,305	9,889

*Data were not recorded for 66 specimens; *MRSA, methicillin-resistant *Staphylococcus aureus*.

**Figure 4:**
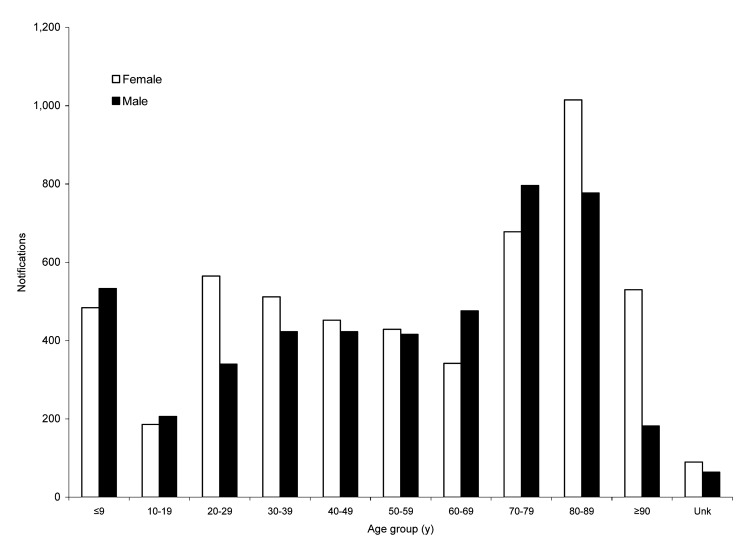
Notifications of methicillin-resistant Staphylococcus aureus in Western Australia, 1998–2002, by sex and age group.

The distribution of MRSA cases from 1998 to 2002 by age and sex is shown in Figure 4. Notification rates peaked in age groups <9 years of age, 20–39 years of age, and 70–89 years of age. Overall, there was a 1:1.1 female-to-male ratio in notifications. In the 20- to 39-year age group, a predominance of female cases was reported; in the 60- to 79-year age group, male notifications predominated. In the >80-year age group, a 61.7% predominance of female notifications was seen; however, this figure was only slightly different than the 64.7% proportion of women in the WA population >80 years of age.

Data were collected on the susceptibility of MRSA isolates to various antimicrobial agents (Table 4). All isolates were susceptible to vancomycin, cotrimoxazole, and clindamycin. Most of the isolates were susceptible to mupirocin. Susceptibility to trimethoprim, tetracycline, and fusidic acid varied, although these antimicrobial drugs remained reasonably active throughout the data collection period. Approximately 40% of MRSA isolates remained susceptible to erythromycin. The only notable change was a significant increase in the resistance of ciprofloxacin, from 11% in 1998 to 26% in 2002 (chi square for linear trend 8.940, p = 0.002), consistent with the increase in numbers of UK EMRSA-15, UK EMRSA-16, and Irish-2 strains. The proportion of multidrug-resistant strains varied from 5.6% in 1998 to 10.4% in 2001.

**Table 4:** Antimicrobial susceptibility of MRSA isolates reported in Western Australia (% susceptible)*

Antimicrobial drug	1998 (1,440†)	1999 (1,058†)	2000 (311†)	2001 (2,326†)	2002 (2,316†)
Gentamicin	98	95	95	92	95
fusidic acid	87	89	87	89	92
Erythromycin	40	43	39	39	40
Mupirocin	99	99	99	99	98
Vancomycin	100	100	100	100	100
Tetracycline	95	94	94	92	94
Rifampicin	99	100	100	99	99
Ciprofloxacin	89	84	76	76	74
Trimethoprim	93	90	93	90	93
Cotrimoxazole	100	100	100	100	100
Chloramphenicol	98	99	99	99	99
Clindamycin	100	100	100	100	100

*MRSA, methicillin-resistant *Staphylococcus aureus*. †Number of organisms tested.

## Discussion

The increasing prevalence of community-associated MRSA is a global public health concern. In WA, colonization or infection with MRSA has been a reportable condition for >20 years, either voluntarily from1983 to 1984 or by law since 1985. This time span has afforded a unique opportunity to document 2 important occurrences, 1) preventing EMRSA from becoming established in the hospital system and 2) emerging community-associated MRSA throughout WA.

The epidemiology of MRSA in WA has always differed from that in the rest of Australia because of the "search and destroy" policy (16,26,27) adopted in the early 1980s. In that decade, the proportion of *S. aureus* that was MRSA varied from 10% to 30% in states other than WA, while WA remained at 0.4% (*17*). After a relatively low number of MRSA notifications in the 1980s in WA, the number increased dramatically in the 1990s. This increase was due almost exclusively to community-associated WAMRSA. The proportion of WAMRSA notifications after 1989 rose remarkably, increasing from 14% to 94% of total notifications in 1998. An almost exponential trend of MRSA notifications was evident, although a possible reporting bias may have occurred as a result of sporadic outbreaks at various times. As is evident from Figure 2, the epidemic of WAMRSA may have peaked; however, several more years of data are required to verify this. Although MRSA now causes 10% of *S. aureus* bacteremia in WA (*28*), the proportion is much lower than that seen in other Australian states (*29*). After 1998, the number of EMRSA notifications in WA started to increase after the introduction of UK EMRSA-15, in particular, and the Irish-2 strains (*22*).

Significant changes in proportions of WAMRSA isolates that occurred in the Perth metropolitan area were not noticeable until 1991, which suggests spread from the remote Kimberley region in the northern part to the southern half of the state. The Kimberley region has a total population of ≈30,000 in a 25,000-km^2^ area. Approximately half the population is indigenous peoples, many of whom live in poor socioeconomic conditions. Infected skin lesions and staphylococcal sepsis occur frequently in this population, and empiric antistaphylococcal therapy is often prescribed (*17*). Some of the disease spread to the south may be attributed to transporting patients from this region to tertiary hospitals in Perth, particularly Royal Perth Hospital, a major trauma center in the eastern metropolitan area. In addition, a large population of "fly-in/fly-out" workers are employed in the area (*14*). Finally, traditionally indigenous populations in Australia are highly mobile.

The highest notification rates of MRSA continued to be in the Kimberley region throughout the study period, which suggested continued involvement of the aboriginal population. However, from 1998 to 2002, the second highest notification rate in the state was the East Metropolitan region of Perth. Historically, Royal Perth Hospital has had more MRSA outbreaks than other Perth hospitals (*14**,**18*), primarily because of the case-mix at Royal Perth Hospital; consequently, Royal Perth Hospital conducts more screening than other hospitals. Until 1997, WAMRSA had not caused outbreaks when patients who were infected were admitted to hospitals, however, during that year an outbreak of a fusidic acid–resistant WAMRSA occurred at Royal Perth Hospital after a patient from another remote rural location (*30*) was admitted. From 1983 to 2002, notification rates increased >50- and 70-fold in rural and metropolitan health regions, respectively.

In 1983, the overall rate of notifications in the rural regions was 10/100,000 compared with the metropolitan area rate of 7/100,000 (*14*). In 2002, notification rates in rural and metropolitan regions were 108 and 104 notifications per 100,000 persons, respectively. In rural regions, the greatest increase in notification rates since 1983 occurred in the Pilbara, Mid West & Gascoyne, and Great Southern health regions with 56-, 48-, and 45-fold increases, respectively. In the metropolitan regions, the South, East, and North Metropolitan notification rates for 2002 were 50-, 23-, and 8-fold higher, respectively, than those reported in 1983.

From 1998 to 2002, 3 peaks in the age and sex distribution of notification rates were apparent: increases in the <9-, 20- to 39-, and 60- to 89-year age groups; male predominance in the 60- to 79-year age group; and female predominance in the 20- to 39-year age group. This female predominance was because the screenees were either nurses on staff or persons who were being screened for potential employment.

Since 1998, notifications as a result of screening increased 4-fold. If the screening tests were removed from the totals, male cases predominated in all age groups. This pattern of age and sex distribution has changed minimally since 1983 (*14**,**20*).

From 1998 to 2002, the proportion of MRSA notifications due to screening increased from 10% to 39%. Screening is still a controversial issue. A recent review concluded that screening of at least high-risk patients was necessary to reduce rates of MRSA infections in hospitals; however, further validation, from a variety of different institutions, of the cost-effectiveness of such programs was suggested and would be valuable (*31*). In WA in 1982, a unified approach to controlling multidrug-resistant MRSA was implemented. The approach was to screen all patients on admission who had been hospitalized or staff members who had worked in a hospital outside WA within the previous 12 months (*17*). Individual hospitals have varied in their approach to controlling non–multidrug-resistant MRSA, which is now endemic in the WA community, but not WA hospitals.

From 1983 to 1992, the agents for which a major change in susceptibility was observed were tetracycline (an increase from <10% to ≈30%), erythromycin (≈10% to ≈40%), and clindamycin (≈30% to ≈80%) (*14*). These changes reflected the increasing proportions of non–multidrug-resistant WAMRSA notifications. In 1993, 68% and 65% of WAMRSA were susceptible to tetracycline and erythromycin, respectively (*8*). The only significant changes from 1994 to 1997 were that fusidic acid resistance increased from 4.6% to 12.4% (*20*), and mupirocin resistance decreased from 6.4% to 0.3% after an earlier high of 18% in 1993 (*32*). Udo et al. first reported high-level mupirocin resistance in WAMRSA strains. This resistance was encoded on a transferable plasmid, which also carried resistance determinants for tetracycline, trimethoprim, and cadmium toxicity (*33*). Mupirocin was used frequently in the northern part of the state to treat infected skin lesions, which resulted in the emergence, selection, and amplification of a mupirocin-resistant strain of WAMRSA. As a result, guidelines restricting the use of mupirocin were implemented; it was not to be used without laboratory control, its use should not exceed 10 days, and >1 month should elapse before further use for the same patient (*32*). After these measures were implemented, mupirocin resistance fell to levels <1% (*32*). This low level of resistance has been maintained for the last 5 years.

Resistance to fusidic acid in WAMRSA continues to be a concern. Resistance has gradually risen from 3% of MRSA notifications in 1993 to 5% in 1994, 9% in 1995, and 12% in 1997 (*34*). From 1998 to 2001, resistance to fusidic acid ranged from 11% to 13% of MRSA, with a slight fall to 8% in 2002. The emergence of fusidic acid resistance in WAMRSA paralleled the decline in mupirocin resistance; some practitioners may have replaced 1 topical antimicrobial drug with another after the guidelines were implemented. The emergence of ciprofloxacin resistance is also of concern. In 1998, 11% of all MRSA isolates were resistant to ciprofloxacin, increasing to 26% in 2002. This change reflects the introduction of UK EMRSA-15, UK EMRSA-16, and Irish-2 into the state (*22*). Ciprofloxacin has been suggested as a possible agent for MRSA decolonization (*35*) and has, at times, been recommended for this purpose by the WA Department of Health. This recommendation may need to be reviewed.

Endemic persistence of MRSA and the measures that should be undertaken to control or eradicate it from hospitals are likely to remain topical subjects. WA has successfully halted multidrug-resistant MRSA outbreaks. The state has seen a persistently low incidence of multidrug-resistant MRSA because a vigilant screening and decolonization program was implemented. During the last 5 years, growth of non–multidrug-resistant, community-associated WAMRSA has been exponential, and rural and metropolitan rates have apparently stabilized. However, new epidemic strains of MRSA, such as UK EMRSA-15, which were seen initially in 1999, steadily increased in 2000 and 2001. The infection control precautions instituted for patients infected with these strains were the same as those with multidrug-resistant MRSA. We do not know whether this approach will work or whether a similar dramatic rise in prevalence of these strains, as seen in UK hospitals recently, will occur (*36*).
